# Maternal haploid waxy maize, environmental effects on grain yield, and quality parameters

**DOI:** 10.1371/journal.pone.0277283

**Published:** 2022-11-10

**Authors:** Rahime Cengiz

**Affiliations:** Field Crops Department, Faculty of Agriculture, Sakarya University of Applied Sciences, Arifiye, Sakarya, Turkey; KGUT: Graduate University of Advanced Technology, ISLAMIC REPUBLIC OF IRAN

## Abstract

Waxy maize (*Zea mays* L. var. *ceratina* Kulesh) is used in the food and textile sectors, amylopectin has an important place in the adhesive and paper sectors as well. These sectors have to buy waxy maize from abroad because there is no waxy maize variety registered yet in Turkey. In vivo maternal haploid technique was applied to obtain doubled haploid (DH) waxy lines in a short time. RWS, RWK-76, and their hybrid RWS × RWK-76 maternal haploid inducers were used as male parents *in vivo* maternal haploid. SSRs markers were used to identify the genetic similarity between the number of 17 DH waxy lines. Similarity ratio ranged from 12% to 68% between DH waxy lines. DH waxy lines were used in crossbreeding and created 24 hybrids. Iodine tests were made on DH waxy lines and their hybrids and analyzed some quality parameters of hybrids. Candidate waxy hybrids were selected from the progeny nursery trial. Several 16 waxy and 3 check hybrids were experimented within three locations and the average grain yield of waxy and check hybrids ranged from 8.4–12.7 t/ha and 12.7–16.2 t/ha respectively. PCA biplot analysis using the data of the average of three locations and genotype × environment interaction was determined. PC1 and PC2 variation percentages were found to be 18.32% and 75.22%, respectively. ADAX-14, ADAX-13R, ADAX-13, and ADAX-19 waxy varieties are more stable in terms of yield than other hybrids. The difference between varieties was found statistically significant for protein, oil, starch, hectoliter, and 1000 grain weight.

## Introduction

Waxy maize (*Zea may*s L. var. *ceratina* Kulesh), possesses 95–100% amylopectin in the endosperm, compared to 70–75% in normal maize. Fresh waxy maize is mainly used as food in Asia and is also an important raw material for food industries, textiles, paper-making, and feedstuff worldwide because of its excellent character in terms of starch composition and economic value. Despite waxy maize being first discovered in China, the origin and evolution of waxy maize are still an enigma [1, 2] China is rich in waxy maize landraces and is considered the source of waxy maize. Waxy gene mutations cause the stickiness of corn grains. Many waxy alleles found in Chinese waxy corn have been identified [3]. Waxy maize is a selective breeding variety with endosperm mutation controlled by the recessive waxy gene (wxwx) and recessive homozygote (wxwx) can lead to almost 100% of amylopectin in the endosperm [4]. The molecular characterization of the waxy locus provides more useful information about the variation of recessive wx1 alleles found in various germplasms. To date, no studies have been conducted to analyze the sequence variation of the entire *wx1* gene. For this reason, the researchers characterized the entire *wx1* allele among 24 different waxy inbreds and determined the number of 29 alleles using SSRs [5].

Starch is traditionally used in the food industry, technological advancement has led to its steady relevance in many other sectors such as health and medicine, textile, paper, fine chemicals, petroleum engineering, agriculture, and construction engineering. It is used in the food industry either as food products or additives for thickening, preservation, and quality enhancer in baked foods, confectioneries, pasta, soups and sauces, and mayonnaises [6]. The basic components of starch granules are two polyglucans, namely amylose, and amylopectin. The molecular structure of amylose is partially simple as it originates in glucose residues linked through α-(1,4)-linkages to long chains with a few α-(1,6)-branches. Amylopectin, the major ingredient, has the same basic structure but has considerably shorter chains and a lot of α-(1,6)-branches. This results in a very complicated, three-dimensional structure, the nature of which remains unclear [7]. The amylose content using modified iodine colorimetry and measured for 454 non-waxy lines and 10 waxy lines with low amylose [8]. They remarked there was a big change in the amylose content. The average grain amylose content was 25.93%, amylose content of waxy lines ranged from 5% to 8%. Researchers point out that the physicochemical properties of corn starch, environmental conditions such as climate, soil, maintenance conditions, and the genetic difference due to variety varies. Sometimes the effect of environmental factors may be greater than the genotypic effect. Many factors, such as including composition, amylose/amylopectin ratio, and granule size, affect the properties of starch [9].

The pedigree method is conventionally used for developing maize lines while selected of plants within segregating F2 populations, making self-fertilization and visual selection at least 7 generations. The average of the population is increased by including the superior offspring obtained from the pedigree method in the next population breeding cycle, which is developed by the recurrent selection method. This obtained population is used as germplasm for the development of inbred lines. Haploid techniques are used to reduce the time required for each cycle and increase the effectiveness of breeding. F_1_, F_2_, or any selected population can be used as donors to obtain DH lines [10]. The ability to produce haploids and then induce whole-genome replication has provided a strategy to significantly speed up the plant breeding process. This method avoids the time-consuming traditional need for intensive selfing or backcrossing during the development of lines used as the parent of the hybrid in maize breeding [11]. In *in vivo* maternal haploid technique, the *R1-nj* color marker carried by the inducer line appears differently in the seeds which are obtained from the induction cross. This color marker allows for determining haploid embryos [12]. Haploid plants include one copy of each chromosome which came from the female parent as obtained from the induction cross of maternal *in vivo* induction. Haploid plants are usually sterile and chromosome doubling is required to produce fertility in haploid plants. Shoot apical meristem contains meristematic cells, which divide and differentiate into organ primordia. To achieve complete fertility in reproductive tissues of haploid plants, chromosomal doubling of meristematic cells should occur before they differentiate into reproductive organs. Therefore, exposing very young seedlings (three to five days after sowing) to mitotic inhibitors is recommended [13]. Colchicine is an effective agent in chromosome doubling [14]. In addition, some of the haploid seedlings die after application, causing the loss of breeding materials [15].

Waxy maize has also been used intensively in recent years for the production of roasted corn snacks and amylopectin starch. There is no waxy maize variety that belongs to domestic or foreign companies registered in Turkey. Waxy maize varieties should be developed to produce the waxy grain that country needs. For this reason, the breeding program was launched for the development of waxy maize varieties in 2011.

## Material and methods

### Production of haploids and haploid seed identification

In this study, 17 F_1_ commercial varieties were obtained from Spain, and their 15 F_2_ waxy populations were used as source material (FAO 600–650 maturity group) for use in the breeding program. Waxy F_1_ and F_2_ genotypes were used *in vivo* maternal haploid technique as donor and female parent at induction cross for 2 years. RWS, RWK-76, and their hybrid RWS × RWK-76 inducer lines which were bought from the University of Hohenheim were used as a pollinator in *in vivo* maternal haploid technique. Since there are no waxy maize varieties in the country, the most preferred 3 hybrid varieties were used as a check, which have normal dent kernel type, at grain yield trials. *In vivo* maternal haploid technique was used to obtain waxy maize lines in a short time.

*In vivo* maternal haploid technique includes the following steps; i) crossing the donors (F_1_ or F_2_ or population or etc.) as a female parent with the inducer lines as the male parent, ii) selection of putative haploid kernels using a color marker, iii) germination of the putative haploid seeds, iv) chromosome doubling to the putative haploid seedlings, v) growing seedlings in optimum conditions and transferring seedlings to the field, vi) obtain of DH seed by self-fertilization D_0_ plants, vii) recurrent selection of DH lines [16].

The waxy germplasm was the main donor and used as a female parent and planted 4 rows, row length of 5 m. Each inducer line, so that a large number of plants were planted at two different times. Ears of waxy genotype (before silk emergence) and tassels of maternal haploid inducers (50% anthesis) were covered with paper. Every row of waxy genotypes was hybridized with each inducer line and fertilized ears were kept in isolation papers until harvest. Four different types of seeds are obtained from the induction cross. Normal diploid or hybrid seeds; endosperm and embryo have a purple color. Haploid seeds; colorless embryo and purple colorful endosperm. Diploid endosperm seeds; colorless endosperm and purple colorful embryo. Outcross seeds; are colorless in both embryo and endosperm [12]. Putative haploid seeds were selected and identified haploid induction rate (HIR). Haploid induction rate was determined as below:

HIR=(Numberofhaploids/Totalnumberofseeds)×100

### Seedling and artificial chromosome doubling

Putative haploid seeds were treated with a fungicide and germination paper was moistened with 0.05% bleach solution to prevent fungal disease growth. The seeds were placed on the paper intermittently, another paper was covered on it, and it was rolled tightly and tied with a rubber band so that it would not open. The rolled papers were placed vertically in plastic containers with 0.05% bleach solution and kept in the growth chamber at 25°C for 3 days. Seedlings with a root length of 3 cm and coleoptile length of about 1–2 cm are ideal for colchicine treatment. The end of coleoptile and root of seedlings were cut using a sterile blade and treated with 0.04% colchicine + 0.5% dimethyl sulfoxide (DMSO) solution for 12 hours at 20°C. Seedlings were washed and planted on plastic seedling trays and grown until reaching 3–4 leaves in a growth chamber. Following planting in the field, inbreeding was conducted for the fertile D_0_ plants during the flowering period.

### Waxy purity analysis

The iodine test is one of the easiest ways to determine the purity of the waxy. An iodine test was performed according to determine the waxy characteristic of each DH line and hybrid. Waxy purity was determined by counting three replications of 100 kernels, moistening them overnight, and then cutting off a small portion from the crown end of the kernel. The kernels were sprayed with a 0.5% iodine solution. Waxy maize contains nearly 100% amylopectin starch which turns a brownish color temporarily after exposure to iodine. This color conversion is short-lived and not permanent. However, maize that contains amylose starch stains a blue or violet color which remains permanently [17].

### Determination of similarity index of DH waxy lines, generation hybrid, agronomic and agro-morphological traits, grain quality composition of hybrids

SSRs markers were used to identify the genetic similarity between DH waxy lines. Thirty SSRs markers were selected from the MaizeGDB database (http://www.maizegdb.org) to provide good coverage of the entire genome. Capillary electrophoresis (Bioptic Qsep100) was used for PCR analysis and allele sizes were determined using Q-Viewer (Version 2.0) of the same device.

DNAs were extracted using CTAB method [18]. Lyophilized tissue of 100–150 mg was homogenized by TissueLyser (Qiagen, Germany) and incubated at 65°C for 90 min in CTAB buffer (1% CTAB; 1M Tris-HCl, pH 7.5; 0.5M EDTA, pH 8.0; 0.5M NaCl; 14M β-mercaptoethanol). After centrifugation of Chloroform-octanol (24:1), the supernatant was transferred into a new tube and cold isopropanol was added. The DNA was washed and precipitated with ethanol and resuspended in PCR-grade water. The quality and quantity of isolated DNAs were measured by using a NanoDrop Spectrophotometer (Thermo Scientific, USA).

After diluting the DNAs, PCR was carried out with 3 μl of DNA (50 ng ul^-1^), 0.03 μl of 100 μM dNTP, 1.5 μl of 5 × PCR buffer with MgCl_2_, and 0.5 μl of 10 μM of each primer with 0.08 μl of 5 units/μl Taq DNA polymerase (Roche, Germany). The reactions were incubated at 94°C for 10 min. After 35 amplification cycles (2 min at 94°C, 30 s at 50–60°C, and 1 min at 72°C), a final extension at 72°C for 7 min before cooling at 4°C were performed. After the amplification, PCR products were separated by capillary electrophoresis in a QSEP100 DNA fragment analyzer (BiOptic, Inc., Taiwan).

For each SSR marker used in the study, the polymorphism information content (PIC) was determined [19]. The PIC calculated with this method is synonymous with the term “gene diversity” [20]. The individual frequencies of these bands were calculated. In addition, the average number of alleles for each amplified microsatellite, and gene diversity (expected heterozygosity; He) were also determined [21, 22].

Genetic parameters including the number of alleles per all locus, allele frequency, expected and observed heterozygosity rate, and null allele frequency were determined. Microsat software was employed to calculate the similarity index [23]. The distance matrix of the genotypes was created and displayed with the NTSYS-pc software.

Hybrid combinations were planned with DH lines determined to be waxy with the iodine test. DH waxy lines were planted in 4 rows as female and male parents. Fertilization was performed by hand and generated 24 hybrid combinations. maize yield trial was designed in a randomized complete block design with three replications. A trial of hybrid waxy maize varieties was set up for 3 locations at Maize Research Institute-Sakarya, Bahri Dagdas International Agricultural Research Institute-Konya, and Black Sea Agricultural Research Institute-Samsun in 2019. Some important climatic data of the locations are given in Table 1. Although there are differences between locations in terms of precipitation, humidity, and temperature, Sakarya and Samsun locations show similar characteristics in terms of climate data. Full-sib was performed on the 3^rd^ row of each variety in waxy corn plots to eliminate the effect of other cultivars and prevent pollen from getting elsewhere at trial in Maize Research Institute. Waxy purity analysis was done in the kernels taken from these rows.

**Table 1:** Important climatic values for long terms of locations at maize growing season (May to November).

Locations	Average temperature (°C)	Average highest temperatures (°C)	Average lowest temperatures (°C)	Average humidity (%)	Total rainfall in season (mm)	Annual total rainfall (mm)
Sakarya	16.4–24.4	22.3–30.5	12.3–19.4	65–88	382.9	878.6
Konya	13.4–24.1	20.4–31.0	7.0–17.2	32–50	124.6	325.3
Samsun	15.9–24.5	19.4–28.2	12.4–20.7	62–87	322.6	729.7

### Data processing

The results obtained from the yield trials of waxy maize candidate cultivars were subjected to analysis of variance using the MstatC [24] program. Duncan’s test was used to determine whether the difference between the means for the yield component was significant [25]. Biplot (G × E interaction) model analysis was performed using the SPSS-16 package program [26].

## Results and discussion

According to the criteria given in the method, putative haploid seeds were selected within seeds obtained from the induction cross. F_1_ genotypes showed HIR in the range of 6–14% while the F_2_ genotypes showed HIR in the range of 5–12% (Table 2). The HIR values of haploid inducer lines were reported as 7.2–14.5%, 1.3–10.2%, 6.4–13.1%, and 8.2–15.54%, respectively [27–30].

**Table 2:** HIR in total seeds from induction hybridization from F_1_ and F_2_ genotypes.

Inducer genotypes	HIR %	
	F_1_	F_2_
RWS × RWK-76	14	12
RWS	6	5
RWK-76	7.2	6.1
Average of HIR	9.1	7.7

**Fig 1:**
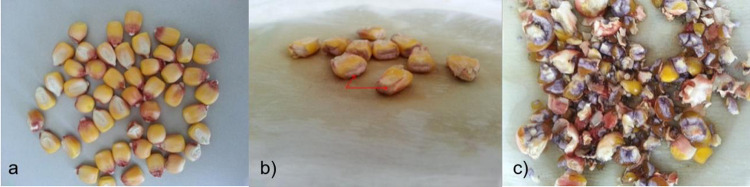
a) Waxy maize grain before iodine test; b) Brownish coloration in endosperm after iodine test; c) Formation of blue-purple color after iodine application in normal maize divided grains.

Chromosome doubling was performed according to the method. Seedlings were transferred to the field and necessary fertilization, irrigation, and weed control were performed. Self-pollinating was made manually when D_0_ plants came into flower. Most D_0_ plants had tassels were produced small amounts of pollen or had anthers without pollen. In the first generation of DH lines, a small amount of grain is formed in the cob because the plants are weak, ear and tassel flowering mismatch and the amount of pollen is low. For this reason, several 17 DH waxy lines were planted the next year to reproduce seeds. An iodine test was conducted to determine the waxy characteristics of each DH line. Amylopectin starch turned into a brownish color immediately after the application of iodine (Fig 1). This color conversion was short-lived and not permanent. Amylose turned into a purple or blue color with iodine application and this color change was permanent (Fig 1).

**Fig 2:**
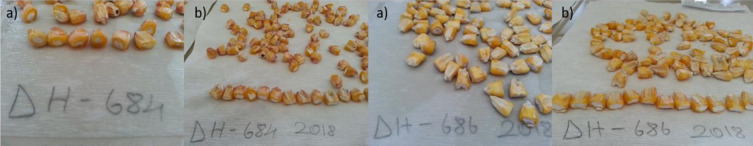
a) Grains of DH waxy line with iodine solution applied; b) Grains of DH waxy line after iodine solution was dried.

If 5% or less of the kernels stain blue or violet, the sample was considered to be waxy; however, it is preferable to have less than 2% of the kernels stain blue or violet [17]. DH waxy lines were pure in terms of this feature and all seeds turned into brownish color and accepted that included 100% amylopectin (Fig 2).

The number of alleles ranged from 2-to 12, and the polymorphism information content (PIC) was determined in the range 0.13–0.70 (Table 3).

**Table 3:** SSR primers to be used to identify genetic similarity, number of alleles, and PIC value.

No	SSR markers	Located chromosome / Segment	Number of alleles	PIC value
1	phi011	8.08	4	0.42
2	phi032	9.04	3	0.48
3	phi050	10.03	5	0.60
4	phi064	1.11	8	0.61
5	phi072	4.01	5	0.63
6	phi093	4.08	4	0.55
7	phi109188	5.03	5	0.59
8	phi109275	1.03	7	0.65
9	phi96100	2.00	7	0.63
10	phi96342	10.02	3	0.49
11	phi402893	2.00	7	0.52
12	phi420701	8.00	6	0.66
13	phi328175	7.04	6	0.66
14	phi299852	6.07	12	0.70
15	phi233376	8.09	6	0.43
16	phi085	5.06	8	0.70
17	phi053	3.05	5	0.66
18	phi070	6.07	4	0.70
19	phi109642	2.04	4	0.39
20	phi002	1.07	4	0.36
21	phi041	10.00	7	0.54
22	phi011	1.09	4	0.42
23	phi033	9.01	7	0.13
24	phi034	7.02	4	0.56
25	phi213984	4.01	2	0.37
26	bnlg490	4.05	6	0.70
27	bnlg105	5.03	6	0.69
28	bnlg1346	5.07	3	0.66
29	umc1225	5.08	4	0.65
30	phi046	3.08	3	0.66

**Fig 3:**
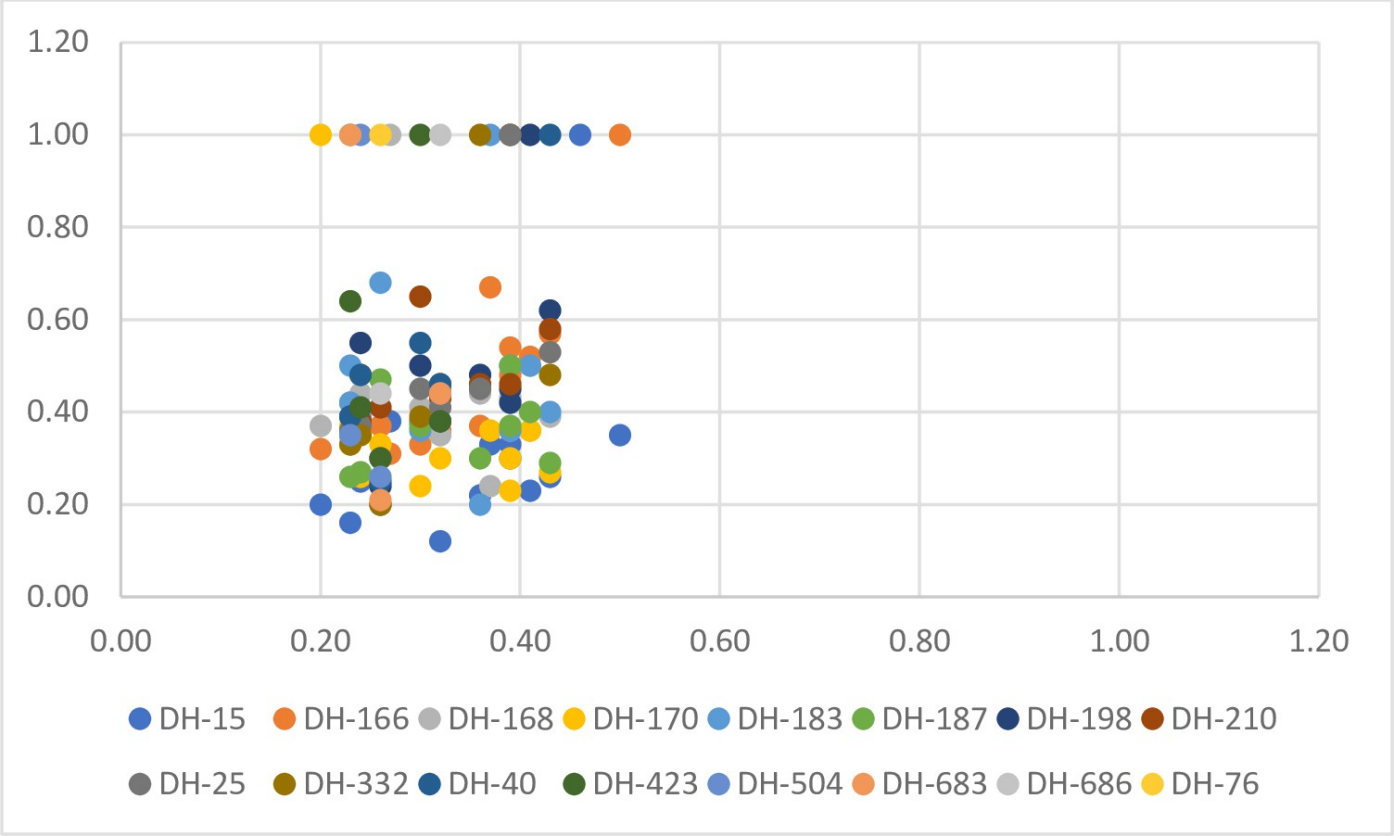
Distribution of DH lines according to similarity index.

The similarity index was created using the Euclidean similarity index. Similarity ratio ranged from 12% to 68% between DH waxy lines. The similarity rates of DH waxy lines accumulated between 20 and 45% (Fig 3). When we evaluate the source material considering the similarity rates, we can say that the F_1_ and F_2_ we use are genetically different from each other.

**Fig 4:**
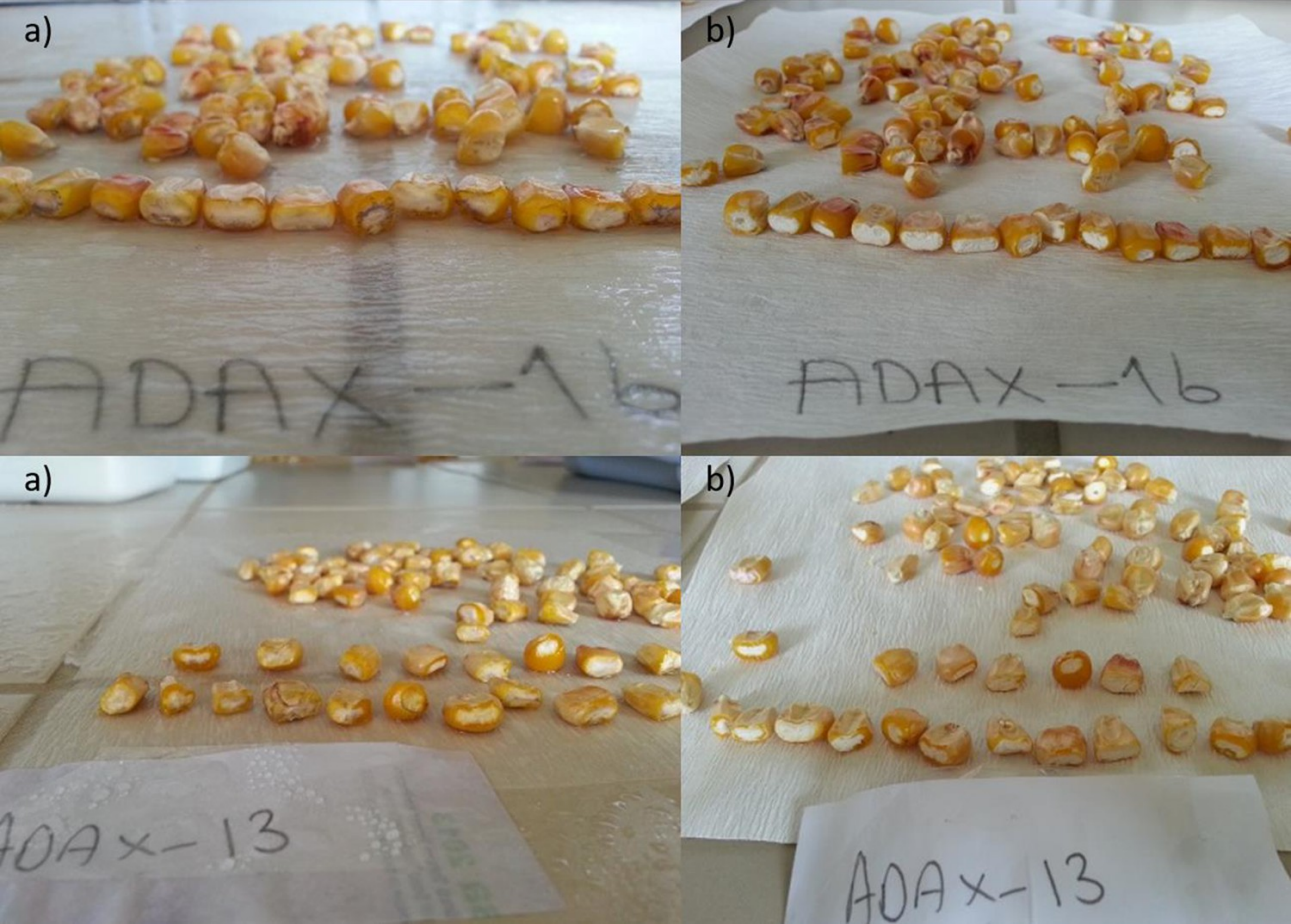
a) Grains of waxy hybrid with iodine solution applied; b) Grains of waxy hybrid after iodine solution was dried.

Female and male waxy parents to be used in crossbreeding were selected considering similarity ratio and features such as plant structure, length, the diameter of ears, tassel structure, and ear size. DH waxy maize lines were planted and hybridization was performed manually and obtained the number of 24 hybrid combinations. Candidate waxy hybrids of 16 numbers were selected from the progeny nursery test. Yield trials were established with 16 waxy hybrid and 3 check dent hybrid varieties. Full-sib was performed on the 3rd row of each variety in waxy corn plots and waxy purity analysis of hybrids was done in the kernels taken from these rows. We considered that waxy varieties were pure in terms of this feature at least 98% turned into brownish color (Fig 4).

The difference between varieties, locations, and variety at the location was found statistically significant for grain yield (P<0.01) (Table 4). According to the average data of the three locations, ADAX-14, ADAX-19 and ADAX-13 waxy varieties came to the fore with their high yields of 12.7, 12.0, and 11.6 t/ha, respectively (Table 5).

**Table 4:** Estimation of significant level for yield of waxy maize hybrids revealed by ANOVA.

Source of variation (SOV)	Degree of freedom (DF)	Sum of squars (SS)	Mean of squars (MS)	F	Probability (P)
Locations (L)	2	45.9490	22.9745	11.2823	< .0001
Reps (Location)	6	18.1742	3.0290	1.4875	0.1892
Genotypes (G)	18	741.7239	41.2068	20.2358	< .0001
L × G	36	403.9803	11.2216	5.5107	< .0001
Error	108	219.9242	2.0363		

**Table 5:** Grain yield results of waxy maize hybrids at three locations.

Varieties	Sakarya loc. grain yield (t/ha)	Samsun loc. grain yield (t/ha)	Konya loc. grain yield (t/ha)	Location average yield (t/ha)
ADAX-2	6.2 h	10.7 d-g	8.2 g	8.4 h
ADAX-8	9.4 ef	12.7 cd	9.5 efg	10.5 ef
ADAX-9	8.2 fgh	10.0 e-h	8.6 fg	8.9 gh
ADAX-9R	8.8 e-h	7.9 hı	8.5 fg	8.4 h
ADAX-11	9.3 ef	10.5 d-g	11.3 b-e	10.3 ef
ADAX-11R	8.8 efg	11.2 d-g	9.3 efg	9.8 fg
ADAX-12	9.9 def	10.5 d-g	9.4 efg	9.9 fg
ADAX-13	12.5 bc	12.7 cd	9.5 d-g	11.6 cde
ADAX-13R	10.6 b-f	12.1 c-f	12.0 abc	11.6 cde
ADAX-14	12.2 bcd	13.7 bc	12.2 abc	12.7 c
ADAX-15R	6.3 gh	10.0 fgh	7.6 g	8.0 h
ADAX-16	10.2 c-f	10.9 d-g	8.4 fg	9.9 fg
ADAX-17	8.4 fgh	7.5 ı	10.6 c-f	8.8 gh
ADAX-17R	8.6 fgh	9.0 ghı	12.2 abc	9.9 fg
ADAX-18	9.8 def	12.5 cd	11.8 bcd	11.4 de
ADAX-19	11.2 b-e	12.2 cde	12.5 abc	12.0 cd
Check-1	18.1 a	11.6 c-f	13.0 ab	14.2 b
Check-2	13.2 b	17.6 ab	7.4 g	12.7 c
Check-3	18.9 a	15.6 a	14.1 a	16.2 a
Significant	**	**	**	**
Average	10.9	11.5	10.3	10.8
LSD (t ha^-1^)	2.60	2.20	2.25	1.33
CV %	14.90	11.57	13.19	13.21
	Location**		Location × Variety**	

**–significant at P≤0.01

*–significant at P≤0.05.

Non-waxy dent check varieties in the trials are the preferred varieties in the country with their high yields, and the waxy candidate varieties did not exceed the yield of these varieties but we think that the yield may be sufficient and the farmer may prefer them. Because will be paid a higher price for the specific grain type. Protein, oil, starch, hectoliter, and 1000 grain weight were determined in the samples taken from the full-sib cobs in the 3rd row of each variety in the trial. Some quality parameters of waxy and check hybrids were given in Table 6. The difference between varieties was found statistically significant for protein, oil, starch, hectoliter, and 1000 grain weight (P<0.01).

**Table 6:** Some quality parameters of waxy and check hybrids at Sakarya location.

No	Varieties	Crude protein (%)	Crude oil (%)	Total starch (%)	1000 grain weight (g)	Hectoliter (kg/hl)
1	ADAX-2	9.3	4.80	56.0	234	76.5
2	ADAX-8	9.6	3.01	57.8	388	80.6
3	ADAX-9	10.2	3.33	57.8	318	77.8
4	ADAX-9R	10.0	3.29	57.7	384	78.6
5	ADAX-11	9.3	3.11	60.1	359	80.0
6	ADAX-11R	9.7	3.00	59.6	379	80.7
7	ADAX-12	9.1	3.46	59.2	357	77.2
8	ADAX-13	10.2	3.42	59.6	347	80.0
9	ADAX-13R	10.6	3.76	58.7	387	78.5
10	ADAX-14	10.3	3.79	58.7	341	75.9
11	ADAX-15R	10.3	3.23	57.7	331	79.8
12	ADAX-16	10.2	4.02	57.1	346	77.5
13	ADAX-17	10.2	3.46	59.1	377	77.2
14	ADAX-17R	10.2	3.40	58.8	374	77.5
15	ADAX-18	10.5	3.93	57.7	324	80.1
16	ADAX-19	10.0	4.02	58.3	321	77.8
17	Check-1	8.9	3.51	60.2	383	78.5
18	Check-2	8.6	3.00	60.6	437	77.2
19	Check-3	89	3.50	59.9	409	79.8
Sign.		**	**	**	**	**
LSD		0.60	0.19	0.97	0.78	0.87
CV %		3.71	3.19	0.99	0.13	0.67

**–significant at P≤0.01

*–significant at P≤0.05

**Fig 5:**
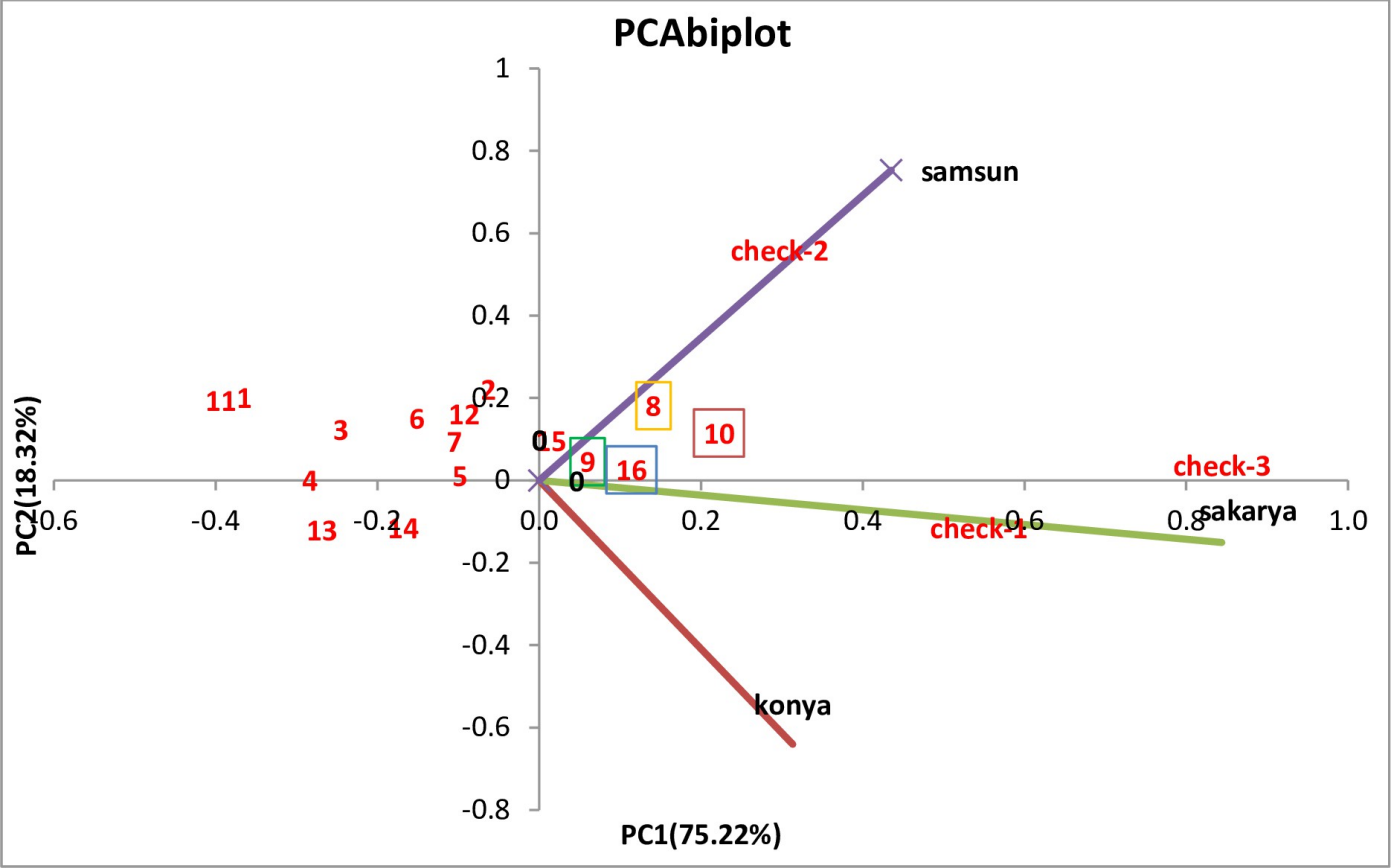
Biplots for combined analysis of 19 maize varieties tested over three locations for grain yield. ^+^1: ADAX-2, 2: ADAX-8, 3: ADAX-9, 4: ADAX-9R, 5: ADAX-11, 6: ADAX-11R, 7: ADAX-12, 8: ADAX-13, 9: ADAX-13R, 10: ADAX-14, 11: ADAX-15R, 12: ADAX-16, 13: ADAX-17, 14: ADAX-17R, 15: ADAX-18, 16: ADAX-19, 17: check-1, 18: check-2, 19: check-3.

The biplot graph describes the values of the two axes of the genotype × environment interaction as a percentage. PCA biplot analysis using the data of the average of three locations is shown in Fig 5. PC1 and PC2 variation percentages were found to be 18.32% and 75.22%, respectively. The graph shows which genotype is more suitable for which environment and reveals the compatibility rates of genotypes for the environment. We can say that the varieties of ADAX-14, ADAX-13R, ADAX-13, and ADAX-19 that were closest to the center on the biplot graph were located better than the others. We can state that the genotypes in the center of the biplot graph are more stable than the genotypes away from the center of the biplot (Fig 5).

## Conclusions

The results in this study of the in vivo maternal haploid technique, which shortens the duration of maize breeding and increases its efficiency, showed that this technique can be easily used in waxy source populations. Genetic and morphological differences between the obtained waxy maize lines allowed hybrid combinations to be made. The fact that waxy hybrid candidates showed lower yields than non-waxy hybrid check cultivars in the data obtained from yield trials conducted in multiple locations resulted in the continuation of breeding studies by adding source materials from different origins. Although the yields of waxy candidate varieties are low, the high product price and the demand in the domestic market make us think that these varieties can be preferred for production.

## Supporting information

S1 Data (XLSX)

S2 Data (XLSX)
